# The telemedicine community readiness model—successful telemedicine implementation and scale-up

**DOI:** 10.3389/fdgth.2023.1057347

**Published:** 2023-02-23

**Authors:** Lena Otto, Hannes Schlieter, Lorenz Harst, Diane Whitehouse, Anthony Maeder

**Affiliations:** ^1^ Research Group Digital Health, Technische Universität Dresden, Dresden, Germany; ^2^ Center for Evidence-Based Healthcare, Branch Office at the Medical Campus Chemnitz of the TU Dresden, University Hospital Carl Gustav Carus, Dresden, Germany; ^3^ European Health Telematics Association (EHTEL), Brussels, Belgium; ^4^ Flinders Digital Health Research Centre, Flinders University, Adelaide, SA, Australia

**Keywords:** community, prescriptive maturity model, scale-up, telemedicine, community readiness, design science

## Abstract

To successfully scale-up telemedicine initiatives (TIs), communities play a crucial role. To empower communities fulfilling this role and increase end users' acceptance of TIs, support tools (from now on entitled artifacts) are needed that include specific measures to implement and scale up telemedicine. Addressing this need, the article introduces the Telemedicine Community Readiness Model (TCRM). The TCRM is designed to help decision-makers in communities to create a favorable environment that facilitates the implementation and scale-up of TIs. The TCRM is a practical tool to assess communities**'** readiness to implement TIs and identify aspects to improve this readiness. The development process follows a design-science procedure, which integrates literature reviews and semi-structured expert interviews to justify and evaluate design decisions and the final design. For researchers, the paper provides insights into factors that influence telemedicine implementation and scale-up (descriptive role of knowledge) on the community level. For practitioners, it provides a meaningful tool to support the implementation and scale-up of TIs (prescriptive role of knowledge). This should help to realize the potential of telemedicine solutions to increase access to healthcare services and their quality.

## 1. Introduction

Telemedicine solutions can increase accessibility to and quality of healthcare services, especially in rural and remote areas (1, 2). The term “telemedicine” describes information and communication technologies that support the delivery of healthcare services as well as medical education by health professionals over a (geographical) distance (3). Telemedicine applications range from synchronous teleconsultation between patient and provider; sending computed tomographies from an ambulance to a hospital before patient arrival; or telemonitoring vital signs, e.g., blood pressure (4). While the initial implementation of telemedicine initiatives (TIs) works well in most cases, their scale-up, i.e., their progress from the pilot stage towards reaching an increased number of people (5), has often been unsuccessful (6, 7).

In other theories described as diffusion, the scale-up process depends on the users' decision to adopt a specific solution (8). Telemedicine users and their decisions are influenced by the specific social, legal, or infrastructural environment (9) they are embedded in. One entity that can actively change the parameters of an environment is the community, whether it is a community of place or one of interest (10). In a community of place, a group of people is connected by a shared geographic and social context, e.g., a city or health network. A community of interest is characterized by common interest independent of the people's location or social group, e.g., people who share conditions resulting from the same disease (10, 11). The community can influence various factors of telemedicine implementation and scale-up (12). Therefore, it is essential to understand the factors influencing communities' readiness to implement and scale up TIs. Readiness describes “the relative level of acceptance of a program, action or other form of decision-making activity that is locality-based” (13), i.e., that shares a common context. In this paper, we investigate community readiness by tackling the following research question:

• *How should an artifact be designed to support the implementation and scale-up process of telemedicine initiatives (TIs) in communities?*

The results are reflected in the Telemedicine Community Readiness Model (TCRM), whose design and development follow a design science-oriented process incorporating literature reviews and semi-structured expert interviews. The construction of artifacts is one area of information systems research, whereby an artifact can be, e.g., a model or method, something that “has, or can be transformed into, a material existence as an artificially made object […] or process” (14).

Our research contributes to information systems (IS) research and practice in different ways. First, we consolidate the community-related factors influencing telemedicine implementation and scale-up. Second, we provide and demonstrate an artifact that helps telemedicine researchers and community practitioners to create a favorable environment for TIs.

We structure the paper as follows: After the introduction, the background section provides information and knowledge that informs the design artifact. The method is part of Section 3, where the procedure for designing and evaluating the TCRM is outlined. The TCRM and its building blocks are presented in Section 4. Section 5 summarizes the evaluation results, and the implications for adapting the TCRM are outlined. The paper ends with a discussion of the results, their limitations, and future research.

## 2. Background

The background section provides knowledge about TIs and maturity models required to design the TCRM and offer practical support to its users.

### 2.1. Telemedicine initiatives (TIs)

TIs enable care delivery regardless of location or time and provide a means to overcome healthcare disparities regarding access to healthcare services, especially in rural or underserved areas (3, 15). Current research streams on telemedicine range from investigations that put the individual at the center of research (16) to studies that highlight the complexity of influences on, e.g., the scale-up and sustainability of healthcare technologies by pointing to the importance of readiness for change or the broader societal system (7, 17). Communities can be placed within the latter. Few studies (9, 18) explicitly examine the influence of communities on the implementation and scale-up of TIs. Our approach follows this stream. We focus on the role of communities in affecting aspects that positively influence TI implementation and scale-up.

**Figure 1:**
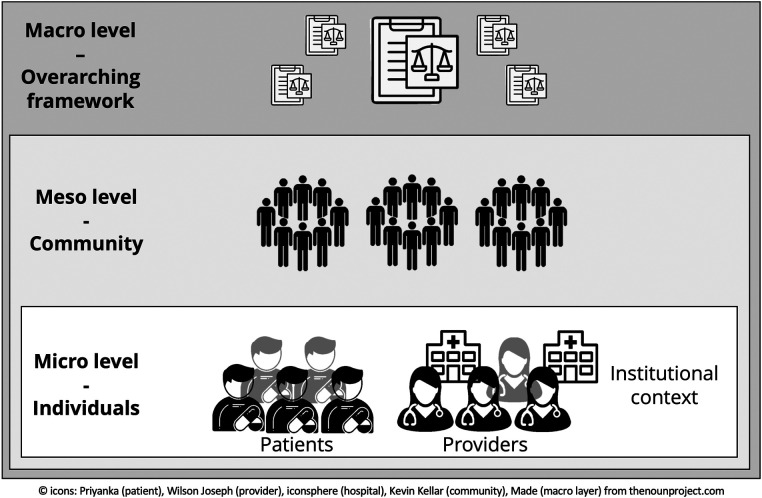
The wider societal context surrounding TIs.

Despite the availability of a variety of technological solutions, studies (9, 19, 20) show that factors like the acceptance by users (e.g., patients or healthcare providers) and their social, technical, or legal environments can influence the implementation of TIs. Figure 1 illustrates the relationship between these aspects and highlights the broader societal context of TIs on the micro, meso, and macro levels (9, 12).

Individuals such as patients and professionals, in their various institutional environments, constitute the micro level of the context around the TI: they decide whether to adopt the TI or not. This decision is influenced, e.g., by the individual's motivation, the usability of the technology, and the organizational regulatory framework in the institutional environment (20). On the macro level, which reflects the overarching framework, the legal and regulatory constraints are defined, e.g., by a federal government or health insurance schemes/companies that define standards and quality guidelines to be followed or funding and reimbursement opportunities (21). Between the micro and the macro levels, on the meso level, the community is located. It represents the social system surrounding the individual. As the users of TIs are locality-dependent, they can be influenced by actions on the meso (community) level.

According to Edwards et al. (10), we understand a community as one of place or one of interest. Other types of communities, such as communities of practice or virtual advocacy groups (22), are not within the scope of this paper. In a community of place, a group of people is connected by a shared geographic and social context, e.g., a city or health network. A community of interest is characterized by common interest independent of the people's location or social group, e.g., people who share conditions resulting from the same disease (10).

There are various influences on communities, depending on their nature. The community is generally affected by macro-level constraints, as it acts within the boundaries of the countries` or systems` overarching framework. The individuals also characterize it on the micro level, which forms the community: the community can affect and support these people by providing them, e.g., with financial and human resources (23), conducting campaigns for raising awareness, or diminishing existing inequalities (18) by setting up support programs, e.g., for financially disadvantaged community members.

### 2.2. Maturity models

In IS research, maturity models (MMs) are used as tools to assess the current situation of the subject under study and further improve this situation by indicating a path for scale-up (24). A certain number of levels typically characterizes MMs (e.g., initial, defined, managed). These levels show a simplified evolutionary path to reach higher “maturity” (25, 26). The levels are accompanied by dimensions, describing activities or key elements relevant at each level (25). MMs are investigated and classified differently, e.g., regarding possible development methods (24, 26, 27), maturation paths (25), or the level of support the MM provides (27). When individual scores can be assigned to different activities on a level, the model's maturation path is called “continuous”, while “staged” models describe the performance of all activities in a single inclusive level (25). Regardless of the specific characteristics used, MMs help different stakeholders to collaborate by providing a common domain understanding (28).

According to de Bruin et al. (27), three types of MMs can be distinguished: descriptive, prescriptive, and comparative. Descriptive MMs consist solely of a description of the status quo. Beyond that, prescriptive MMs include recommendations for possible improvement steps. When sufficient data is collected to benchmark, the model can become a comparative MM enabling the comparison of various industries or regions (27).

MMs have already been applied in the field of telemedicine. An example is the descriptive model of (29), who present a 5-point, Likert-like questionnaire, which focuses on hospital staff as end users. However, the model lacks clear documentation on how to apply it. Additionally, Likert-like questionnaires are generally intensely subjective as no information is given for each score (25). Another example is the maturity grid of (30), which is also descriptive but omits a clear statement of who the target audience is and lacks a focus on the community perspective (31). It was further developed by the same authors (32) into a more substantial but even more complex MM, which makes it hard to easily understand and use it (33).

An existing MM that considers the influence of communities is the Community Readiness Model (CRM) for prevention programs (10, 34); it concentrates on community efforts or the community climate (34). Since telemedicine specifics, such as the focus on technical infrastructure and support when using digital solutions, are not part of the CRM, they cannot be applied directly to TIs but are used as a basis for considering the community.

In summary, existing telemedicine MMs either focus on specific aspects of telemedicine (leaving aside the complexity of the context and the supportive role of communities) or have some shortcomings regarding the support of the improvement process (12). However, MMs are a promising tool to guide implementation and scale-up processes (30). As existing approaches are not sufficient, the TCRM combines a staged (type of MM), prescriptive MM approach (guidance character) with both TI and community characteristics (scope).

## 3. Method

**Figure 2:**
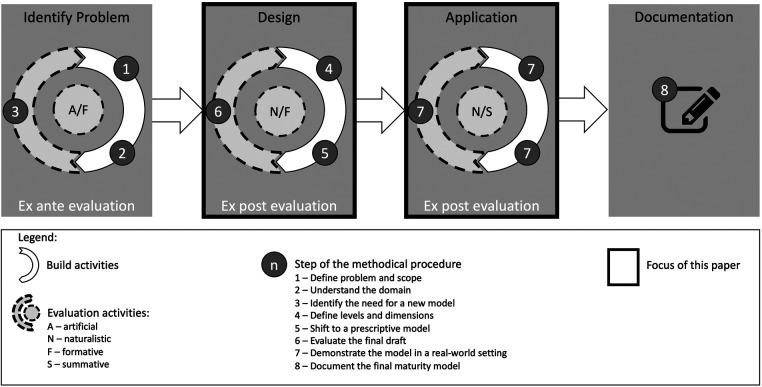
Methodical process to design the TCRM (refined research process based on (35).

Inspired by the MM development procedure of (24), we conducted eight steps that implement a specific procedure along these cycles. This paper documents the design and application of the artifact (second and third cycles indicated by a black frame in Figure 2).

### 3.1. Prior research and problem justification

Our research addresses the problem (step 1) of the slow implementation and scale-up of TIs worldwide (36), and the context-sensitivity of Tis (37). To understand the domain (step 2), community-specific (10) and telemedicine-specific barriers and enablers were analyzed based on two literature reviews [see the previous study by (12)]. For example, while missing collaboration culture or lacking knowledge about the existence and use of TIs can impede the implementation and scale-up of telemedicine, the provision of adequate resources and the involvement of qualified stakeholders can enable this (12). The need (step 3) for a prescriptive MM focusing on the community was shown by (31). Having investigated eight prior MMs, including their shortcomings, they conclude that a new MM for telemedicine should incorporate elements such as “community”, core readiness, barriers, and adequate guidance materials (31). All these elements were included in the TCRM to provide an artifact advancing the field of MMs for TIs, ready to be applied.

### 3.2. Designing the TCRM

The levels and dimensions of the staged TCRM (step 4) are inspired by other MM approaches (10, 20, 30, 34) and integrate knowledge about evidence for TIs (38–40). After combining levels and dimensions in analogy to existing models (e.g., van Dyk and Schutte 2012), barriers and enablers for implementing and scaling-up TIs (12) were added to the maturity levels. The model got a prescriptive character by including advice on evolving towards a higher maturity level (step 5).

### 3.3. Demonstration and evaluation of the TCRM

According to (41), MMs are seen as in-between the artifact types model and method. Demonstrating and evaluating a MM should therefore consider the evaluation criteria for models and methods alike (41). 17 qualitative, semi-structured expert interviews addressed these evaluation criteria (see Table 1) to reflect the TCRM with potential key users (steps 6–7). The interviews were conducted in two rounds (first round: 12 interviews, second round: 5 interviews) with experts in Australia and Germany, and are further described in Section 5.

**Table 1:** Evaluation criteria for models and methods.

Round of evaluation (by expert interviews)	Object of evaluation (42)	Evaluation criteria (43)	Artifact type (43)
First (Section 5.1)	Structure	Completeness	Model
		Fidelity with real-world phenomena	
		Internal consistency	
Second (Section 5.2)	Goal and environment	Level of detail	Method
		Generality	
		Ease of use	
Long-term	Evolution and activity	Efficiency	Model
		Operationality	
		Robustness	

## 4. The telemedicine community readiness model (TCRM)

The TCRM consists of three parts: An **assessment part** to define the current readiness, an **improvement part** (as it is a prescriptive MM) that helps communities shift to higher levels, and a **procedure model** guiding the use of the model.

### 4.1. How to assess communities with the TCRM?

According to (44), the scope of a MM needs to be defined by describing the model's focus, its target audience, and the relevant stakeholders. As shown in Table 2, the TCRM focuses on communities of place and/or interest, the target audience are decision-makers in a community (also called multipliers). Still, we underline the advantage of involving experts with a heterogenous background.

**Table 2:** Scope of the TCRM.

Focus	Target audience (people who are interested in the results)	Relevant stakeholders (people able to assess the as-is situation)
- Telemedicine readiness in communities ○ of place (e.g., a municipality, region, or healthcare network) or ○ of interest (e.g., a network of patients with a particular disease) or ○ a combination of both (e.g., people with a certain condition within a geographic boundary)	- Decision-makers on an administrative level (e.g., representatives in communities, payers, legislative institutions or service providers)	- All people within the community involved in managing, delivering, and using telemedicine, e.g., healthcare professionals, technicians, or patients - Ideally, different experts from various backgrounds will together apply the TCRM to ensure the reliability of results

**Figure 3:**
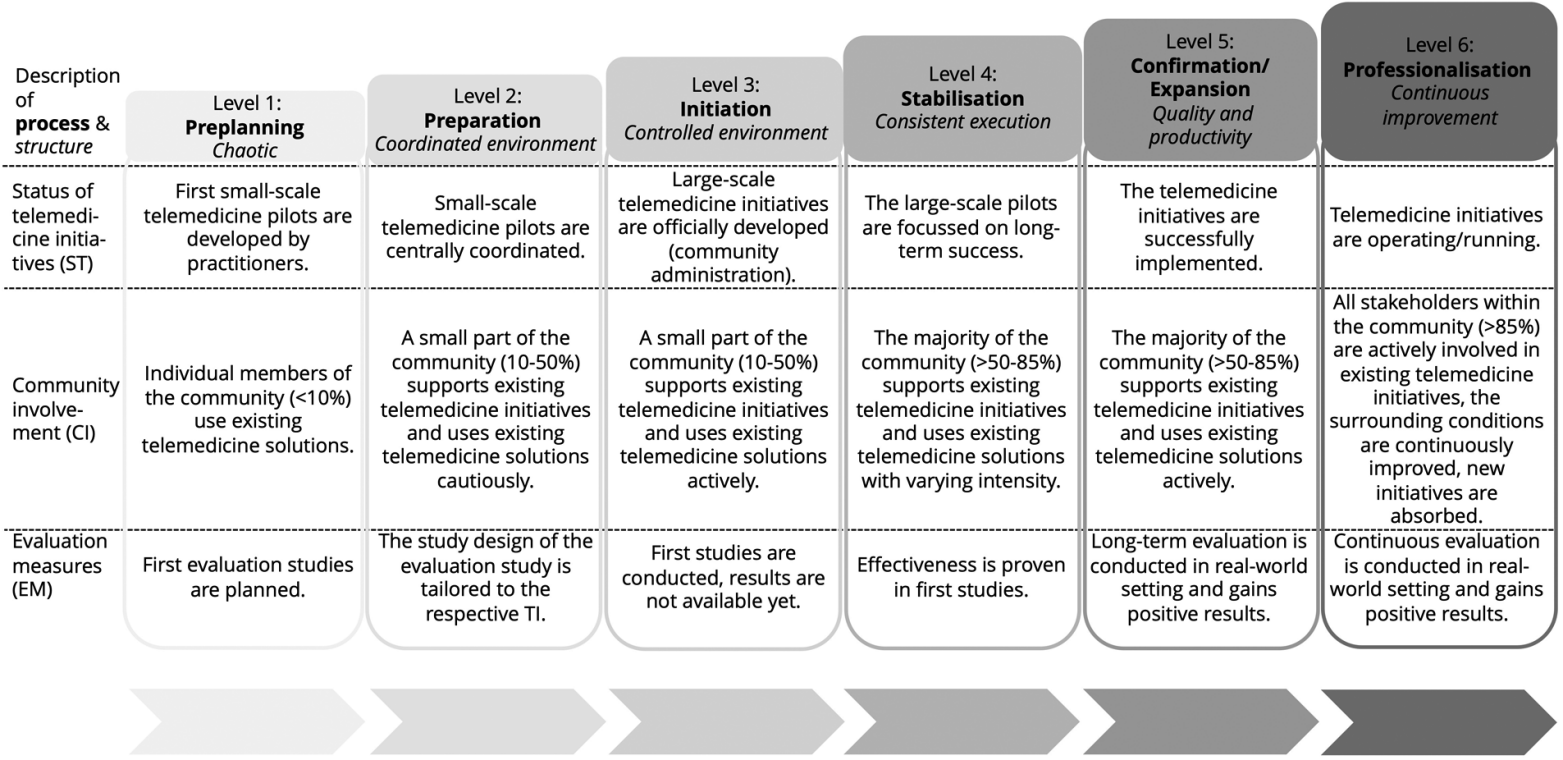
The assessment part of the TCRM.

The TCRM depicted in Figure 3 includes all the factors related to and influenceable on the community level (vertical). For example, the individual's unwillingness to use telemedicine should be addressed on the micro level, while regulatory issues have to be dealt with on the macro level.

In the TCRM, three dimensions (status of telemedicine (ST), community involvement (CI), and evaluation measures (EM)) and six levels structure the assessment part (Figure 3). Process-related and structural descriptions characterize the six levels. The levels describe an evolutionary path towards successfully implemented and scaled-up TIs, where all levels need to be reached consecutively. Thus, the TCRM is additive, i.e., every aspect considered at a lower level also needs to be fulfilled at all subsequent levels. To illustrate the components of the model and their interplay, we use the following two examples.

Example 1—Progress from Level 1 (Preplanning) to Level 2 (Preparation): At Level 1, the environment for implementing TIs in the community is *chaotic*, i.e., there is no structure provided to the community. Only a small proportion of the community members (e.g., patients, other citizens, or healthcare providers) participate in sporadically developed telemedicine pilots (e.g., applications are tested in only one hospital). No empirical evidence has been gathered, but initial evaluation studies have been planned. When the environment becomes more *coordinated*, i.e., the community starts to take responsibility for coordinating the development strategy for TIs, community readiness evolves toward the second level. The number of community members using the existing initiatives increases, but the solutions do not convince everyone. Evaluation studies are now designed to incorporate the needs of the individual TI.

Example 2—Progress from Level 5 (Confirmation/Expansion) to Level 6 (Professionalization): At the fifth level, where the focus is on *quality and productivity*, most initiatives are completed successfully (e.g., applications are implemented for all the potential patients in the community). The majority of community members actively use TIs. Existing TIs are expanded to other diseases or community members who are accustomed to using TIs. Evaluation activities are steadily conducted in real-world settings, and positive results are gained in the long term. When the focus shifts to *continuous improvement,* this indicates the sixth level: TIs are established in the community and regularly maintained and improved as a joint initiative among all stakeholders involved. Almost all community members have access to TIs and use them. New initiatives can easily be included and are available to all community members. The evaluation activities are conducted in real-world settings to generate constant evidence about the TIs.

### 4.2. How to improve guided by the TCRM?

**Figure 4:**
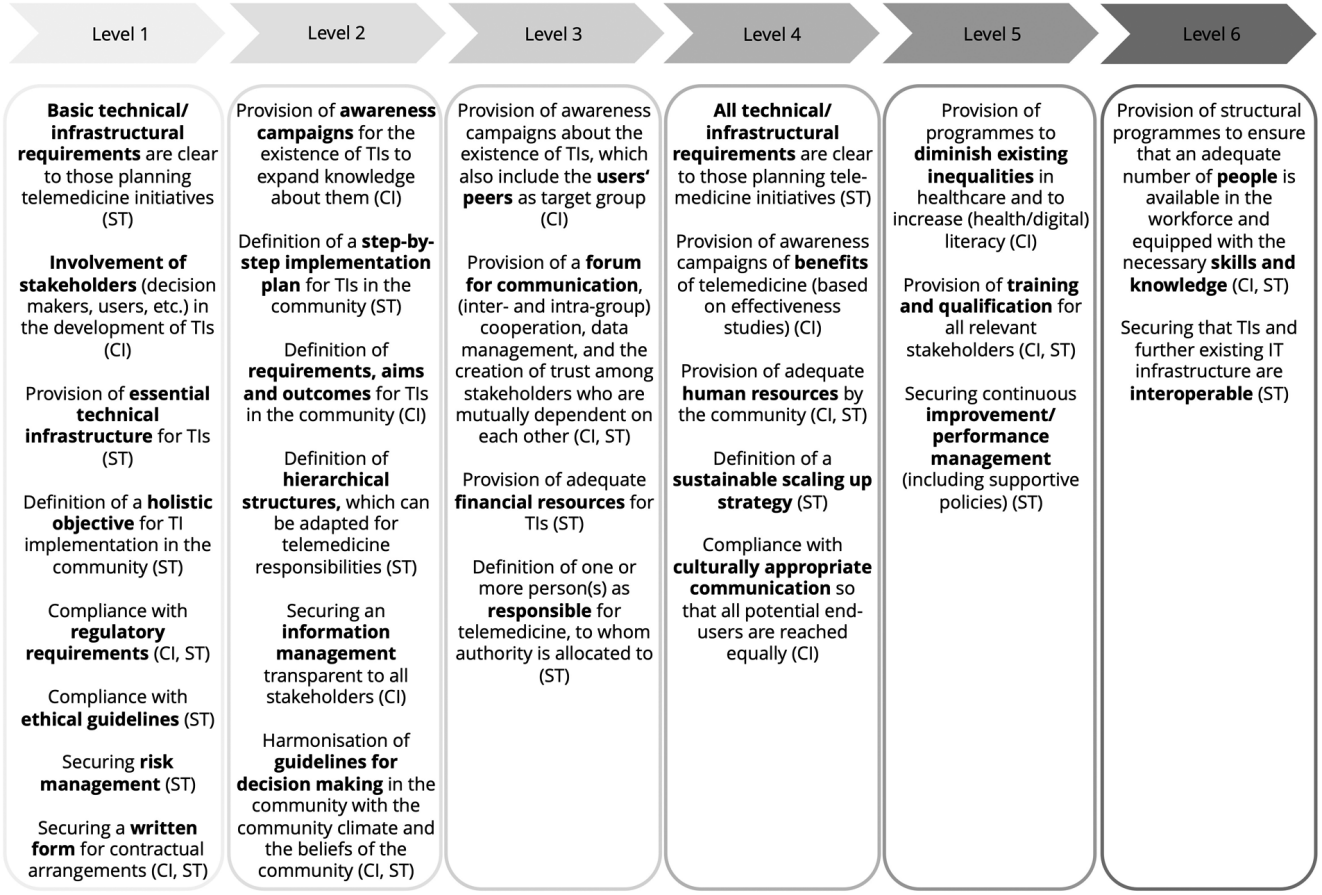
The improvement part of the TCRM.

The TCRM also provides improvement aspects (Figure 4) that support moving from lower to higher maturity levels. Each improvement aspect (see Supplementary Annex A1) influences the status of TIs (ST), community involvement (CI), or both, as shown in brackets for each aspect in Figure 4. To illustrate the improvement process, the two examples cited above are used again.

**Example 1**: Aiming to progress from the first to the second level, community actors should consider all aspects on the first level and monitor their fulfillment. For example, a community can have a *holistic objective* to implement telemedicine, the *basic infrastructural requirements* are clear, and *essential infrastructure* is provided. Furthermore, *risk management* is ensured, and *contractual arrangements* are documented in written form. Based on this initial situation, the community should ensure that all the relevant *stakeholders (especially patients) are involved* when implementing new TIs. The community also needs to be aware of existing *ethical guidelines* and guarantee they are continuously followed. When these measures are implemented to fit the community's needs, this community is ready to advance to the second readiness level.

**Example 2:** To progress from Level 5 to Level 6, the respective improvement aspects on Level 5 need to be considered. A community at the fifth level could have supportive policies in place to ensure *continuous improvement/performance management*. As a next step, *programs should be set up to support increasing the (health/digital) literacy of community members and diminish any inequalities* in the community. Measures for *training and qualification* need to be provided permanently to ensure that all relevant stakeholders can adequately use TIs and help others to do so. As the model is additive, the improvement aspects depicted in Levels 1 to 4 need to be monitored continuously. Accordingly, in case of maturing from the first level onwards, all improvement aspects can be assumed to have been considered. In case the initial assessment of the current status of community readiness results in a higher level, such as the fifth level, the fulfillment of all improvement aspects on the previous levels needs to be checked. If, for example, the community has already implemented awareness campaigns but did not include users' peers as a target group, this aspect needs to be worked on. After that, it is possible to progress to the sixth level.

### 4.3. How to apply the TCRM?

**Figure 5:**
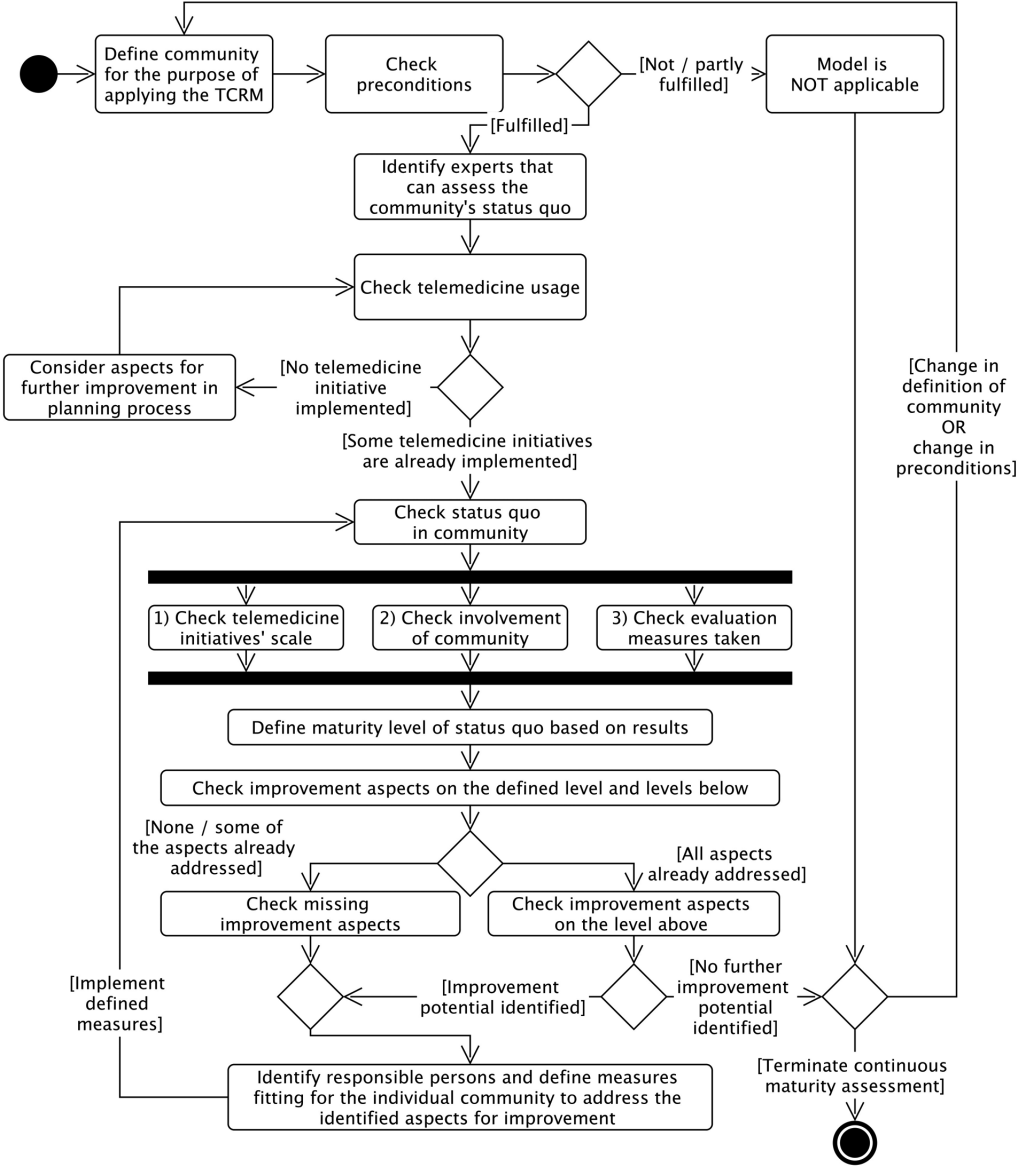
Procedure model of the TCRM.

The procedure model of the TCRM (Figure 5) describes the detailed activities and decision points that should be dealt with during the use of the TCRM. This assures compliance with the intentions and mechanisms of the TCRM.

First, the community (of place and/or interest) to which the TCRM shall be applied has to be defined. Afterwards, the applicability of the model is checked based on two preconditions to be fulfilled:

1. In the community exists a core readiness for change, which means there is a common desire to use TI and to change traditional healthcare processes (18, 45).
2. The community has implemented or started implementing at least one TI. (In the case that no TI is implemented, the improvement aspects in the model can still be used during the telemedicine planning process.)

When the preconditions are fulfilled, experts (external auditors or community members) have to qualitatively assess the as-is situation regarding the three assessment dimensions (ST, CI, EM). In the case that the individual assessments for the dimensions differ, the following rules apply:

Generally, the lower rating of the two ratings of ST or CI should be taken as the overall rating, representing the dimension that must be improved first. For example, if the ST is on Level 3, the CI on Level 2, and EM on Level 3, the (telemedicine) community readiness is at Level 2. Lower ratings of EM are not directly included in the assessment as evaluation measures can be initiated directly. For example, if ST and CI are on Level 3 but EM is on Level 2, the overall rating is Level 3, with evaluation measures being one of the improvement aspects to reach higher levels. The same applies if no evaluation studies are planned. A rating cannot be given in that case as EM is below Level 1. Since the other two dimensions need to be at least on Level 1 (as one TI must be implemented already according to the preconditions), the overall readiness of the community would be at Level 1, with EMs being the most important aspect to focus on.

Afterward, all improvement aspects described on the rated level and the levels below need to be checked to judge their fulfillment. If all aspects are addressed, the aspects of higher levels can help identifying measures for improvement. Whenever improvement potential is identified, the responsible persons who can guide the improvement process need to be identified to help implementing the improvement aspects with measures that fit the specific community characteristics. Persons who may offer guidance include professionals, technicians, or other users of TIs. Once all improvement measures have been implemented, the process can be repeated for continuous improvement. Undertaking this action supports the scale-up of the ST and the CI and helps the community moving to higher levels of TI readiness.

## 5. Evaluation

The TCRM was assessed regarding its structural characteristics, goal and environment (42). To assess these characteristics, two rounds of qualitative, semi-structured expert interviews with potential user groups were the most feasible approach. In the first round, the focus was on the structure; in the second round, the focus was on the goal and effectiveness of the TCRM, which was then modified based on the experts' feedback.

### 5.1. Evaluating the TCRM's structure

The first version of the TCRM was evaluated with twelve experts to obtain their opinions on the *completeness*, *fidelity with real-world phenomena*, and *internal consistency* of the model structure to adjust it, if necessary. During the interviews, the “think aloud” method (46, 47) was used to understand the interviewees' impressions of the descriptive model.

The twelve interviews were conducted in Germany (*n* = 7) and Australia (*n* = 5) between March and July 2019. Germany and Australia were chosen as both countries rank similarly on international scales of socio-economic comparability [high-income developed countries (48)] but have substantially different contextual settings. While Australia is characterized by a definite contrast between its urban and rural areas and a National Health Insurance System, Germany is densely populated throughout and has a Social Health Insurance System in place. In Australia, the state is responsible for regulating and financing the healthcare system, whereas in Germany both tasks are carried out by societal actors (49). In both countries, care provision is carried out by private actors. Thus, the TCRM was tested in different environments. The following criteria were applied during the recruitment process: all experts needed to represent members of the target audience or stakeholders for future use of the model (see Table 2). Furthermore, they had to have personal experience in implementing or using TIs in their job. The experts assessing the structure of the TCRM included healthcare professionals, representatives of health insurance companies, and/or representatives of network organizations in healthcare (Table 3).

**Table 3:** Country and expert's status per interview with the structure-interview experts (SIEs).

Country	Germany							Australia				
SIE no. Role	1	2	3	4	5	6	7	8	9	10	11	12
Healthcare professional		x					x			x	x	
Representative of a health insurance company			x			x						
Representative of a network organization in healthcare	x			x	x			x	x	x	x	x

All interviewees stated that the process described by the TCRM's levels and dimensions was similar to their real-world experience in their communities, for example: “it's true, we started off […] in the planning […] phase and then […] we have improved. […] Now we’ve got a few more sites […] and became a bit larger” (SIE11). Some adaptions to the initial TCRM were made to address the experts' feedback. The adaptions concerned the descriptions of levels and dimensions as well as the wording and the assignment of improvement aspects to levels (see Supplementary Annex A2). Each expert was asked to assign each improvement aspect to one of the six levels in the model. The median of this assignment was then calculated across all interview results (see Supplementary Annex A3). Adaptations to the model were made by two authors based on this calculation and the explanations the interviewees offered while thinking aloud. Given the small number of interviewees, extreme median values carry the risk of biased allocations of improvement aspects to the steps of the model. Therefore, all assignments were weighted in line with each interviewee's expertise and her or his statements during the allocation process. For some improvement aspects, the median resulting from the assessment by the Australian and German interviewees differed. As the number of interviews and countries was not high enough to assume that the TCRM needs to be country-specific, this needs to be explored in more detail in future work.

Most interviewees remarked that the model represents an idealistic path to the scale-up of telemedicine. Nevertheless, it is “useful to have an ideal […] model, because in a process where you are guided by it, you do not run the risk of forgetting things that are essential” (SIE6).

### 5.2. Evaluating the TCRM's goal and environment

Revised on the basis of interview round no. 1, the TCRM was afterward applied in real-world settings to ensure that it is understandable to its potential users and can easily be applied. For this, the evaluation criteria were *level of detail*, *generality* and *ease of use* (42). Therefore, five interviews with different experts were conducted in Australia (*n* = 3) and Germany (*n* = 2) later in 2019. Following the selection scheme of the first iteration, healthcare professionals and/or representatives of network organizations in healthcare were interviewed (45). The TCRM was applied by the interviewees to their communities using the procedure model (see Figure 5). This process was supervised by the interviewer to identify weaknesses of the TCRM's documentation (*level of detail*) or the documentation of the process model (*ease of use*).

The communities described by the five experts for applying the model (AIE1—AIE5) varied across states (in Australia) and across different conditions in diverse types of cities (in Australia and Germany). Through this procedure, it could be shown that the TCRM can be applied to communities of place and of interest (*generality*). Four of the five communities were assessed as Level 1 by the experts (Table 4). One community was rated on Level 4. The TCRM could help identifying improvement aspects for each community, e.g., AIE2 stated that “culturally appropriate service response, particularly for the aboriginal community”, is needed, which corresponds to the “culturally appropriate communication” aspect.

**Table 4:** Expert and community characteristics during the second round of interviews with application-interview experts (AIEs).

	AIE no.	1	2	3	4	5
	Country	Australia			Germany	
Role	Healthcare professional	x		x	x	x
	Representative of network organization in healthcare	x	x	x	x	x
Definition of community	State as geographic boundary *(community of place)*	x	x			
	City as geographic boundary *(community of place)*			x	x	x
	Condition *(community of interest)*	Stroke diagnosis	Need to receive rehabilitation	Not specified	Not specified	People living in nursing homes in that city
	**Overall level of readiness**	1	4	1	1	1

The interviewees were also asked to assess the TCRM's usability. AIE3 expressed general doubts as to whether the model provides a one-size-fits-all solution: “I hesitate that the model suits everybody or every condition” (AIE3)). This concern can be addressed by more evaluation activities in the future to find out how universally the TCRM is applicable. However, the model is based on international evidence, e.g., related to barriers for TI implementation worldwide, and it is therefore assumed to be widely applicable for further evaluation.

More detailed feedback was given by the last set of interviewees concerning the suggested improvement activities (“examples would be very helpful” (AIE1), “the contractual arrangements need to be documented in written form” (AIE5)). Wherever possible, the interviewees' feedback was incorporated into the model or in the documentation accompanying it. AIE4 commented that the model is structured in a logical way and can support argumentation with decision-makers. Additionally, AIE2 confirmed the usefulness of the model as “it's been reassuring to know […] the activities of my team […] are wrapped into your model” (AIE2), i.e., the “idealistic” path identified in research points towards the same direction as the activities already conducted in the community of AIE2.

## 6. Discussion

### 6.1. Scientific implications

The TCRM contributes to theory and research by focusing on the role of communities in TI implementation, thereby bridging the gap between individual adoption decisions and a society-wide effort to implement TIs. Communities and their influence on innovation acceptance are also considered in theories and models of behavioral change and technology acceptance (e.g., the UTAUT2 (50) or the Diffusion of Innovations theory (8)). In the context of these models, however, the community is labeled “social influence” as one predictor variable for individual acceptance and adoption of health technologies (51). In contrast, the TCRM places the community on the intermediate (meso) level and thereby mediates between the individual adoption decision and a society-wide decision to implement TIs.

By utilizing the TCRM in five different communities, we showed that the TCRM provides a valuable tool for the maturity development concerning community readiness for TIs. The prescriptive character of the TCRM helps to integrate best practice knowledge as potential measures to improve the situation (27). Having based the suggested measures of the TCRM on barriers and enablers identified in international studies (12), and learnings from the successful application of the CRM (10), an adequate evidence base is ensured.

The focus on the community for successful TI implementation and scale-up also seems feasible. This is underlined by the expert interviewees' feedback and prior literature. In the NASSS framework of (17), for example, the authors recognize the role of communities in the implementation process by considering socio-cultural aspects as part of the wider system influencing an adoption decision. Also (52), considered the community an essential actor when supporting eHealth tools. However, the TCRM goes beyond these approaches by providing an artifact that emphasizes the role of communities and helps empower communities to make a change toward successful TI implementation and scale-up.

Compared to existing approaches, such as the CRM (10), the TCRM also incorporates knowledge about barriers related to TI, such as an absence of infrastructural conditions, interoperability challenges, or health sector barriers such as an inadequately skilled workforce.

Especially the improvement aspects in the TCRM can help defining outcomes to consider when evaluating implementation as suggested in the evidence standards framework for digital health technologies suggested by the NHS England. In this framework, the seamless integration of any healthcare technology into existing processes is considered as basic requirement whose fulfillment needs to be proven in any evaluation process (53).

### 6.2. Practical implications

The TCRM was evaluated during the design and application phase by seventeen expert interviews. The experts confirmed that the TCRM is a valuable artifact to support the implementation and scale-up of TIs and help communities to increase their readiness for TIs. Notwithstanding a longer-term evaluation of the model's effectiveness, it can be assumed that the model helps TIs to move beyond the pilot phase (54).

To appropriately communicate the research results (55, 56) and to ensure the TCRM is accessible and usable by practice, it has been published as an easily applicable online tool free of charge, including an option to provide ongoing feedback^1^. To date, the TCRM has already been used by experts in other countries beyond Germany and Australia, e.g., for one community in Croatia (Level 4), one in Norway (Level 3), and one in the United Kingdom (Level 4). Even though embedded in different healthcare systems, each community could define its readiness status and identify improvement measures. The higher readiness levels in these three countries may indicate that the low levels of readiness in the Australian and German demonstration cases for the TCRM are not representative for the state of telemedicine internationally (57). Benchmarked different countries regarding their digital health index and placed Australia and Germany in groups three and four out of four groups ranking their digital health development, while the Nordic countries or NHS England are placed in the first two groups. Interestingly, even in the same national Australian or German framework, their readiness levels differ, which supports the influencing role of the community regarding TI implementation.

The TCRM will need to be continuously maintained to ensure its ongoing relevance (27). As the TCRM has been applied to communities in five different countries with various settings and is based on international evidence, it can be assumed that the TCRM can be applied in other countries as well. However, more extensive and longer-term evaluation studies would be necessary to prove this assumption. Larger scale application of the TCRM could also trigger macro-level activities, e.g., if communities in specific regions or countries all have lower levels of readiness that could alert a country's policy-makers to adapt legal or regulatory provisions.

### 6.3. Limitations

Our approach comes with three limitations. First, designing the TCRM included subjective decisions. We reduced bias by conducting each step in the design process in pairs of two researchers, except for holding the interviews and real-world application sessions. Inconsistencies were resolved through discussion to reach a consensus. Second, we showed the importance of communities in TI implementation and scale-up and validated the TCRM based on expert feedback. We could not, however, evaluate implemented change measures based on the usage of our artifact. Therefore, the artifact's evolution and activity (42) need further longer-term evaluation. Third, the evaluation of Levels 5 and 6 of the TCRM is limited. The expert group consisted of people who rated their community to be on the first four levels. However, all of them stated that the model could be helpful to further increase the readiness of the communities they represented.

### 6.4. Future research

To further validate the model, more extensive and longer-term evaluation studies with different experts will be necessary to focus on the following four aspects: First, it is essential to evaluate the TCRM's *efficiency*, *robustness*, and *operationality*, i.e., its evolution and activity (42) in the longer-term, as well as, its impact on telemedicine readiness at all. We also assume that there are some constraints related to the type of telemedicine solution, which should be analyzed. Second, more extensive studies could reveal whether country-specifics [e.g. (58),] cultural dimensions) need to be incorporated into the TCRM, which could then lead to the formulation of a comparative maturity model (59). Such a model would enable benchmarking of different regions on a more objective level (27). Third, further demonstration and evaluation activities should focus explicitly on communities of interest. This type of community was, in the current paper, only included in relation to an additionally shared geographic context. Fourth, broadening the range of case examples should also include communities on higher readiness levels to further validate Level 5 and 6. Such studies could also reveal if different kinds of payer systems make a difference in how far the community can support TI implementation. While the improvement aspects in the TCRM represent an impetus for enhancing the current readiness status, each community needs to identify and implement measures that fit its specific context and structure. As a next step, an exchange of best practices between comparable communities would help implementing specific improvement measures—as has been done with similar tools (Grooten et al. 2019).

## 7. Conclusion

The paper was motivated by the need for a community perspective aiming to successfully implement and scale up TIs. We showed the shortcomings of prior research, calling for a suitable tool that addresses the community's readiness to apply and scale-up TIs to provide value for the citizens and decrease disparities in healthcare systems. The TCRM has the potential to develop community readiness and to drive TIs in a direction where they can generate value for the people, which is the central concern of design-oriented research. It can interest payers, healthcare professionals, and key community stakeholders and can be explicitly used in health services research to expand needs analyses. In the sense of an evolutionary concept of design work, we hope the TCRM is seen as a proposal to evolve in the community and to foster the discussion on how we can speed up digital health generally and TIs specifically.

## Data Availability

The original contributions presented in the study are included in the article/**Supplementary Material**, further inquiries can be directed to the corresponding author/s.

## Footnotes

^1^ http://care4saxony.de/?page_id=3837
