# Supplementary material for: The telemedicine community readiness model—successful telemedicine implementation and scale-up
Source: Front Digit Health. 2023 Feb 23;5:1057347. doi: 10.3389/fdgth.2023.1057347 (PMC9995762; doi:10.3389/fdgth.2023.1057347)
Supplement: Supplementary file 1 [file Datasheet1.pdf]

Annex

Annex A1 - Detailed description of improvement aspects

	Improvement aspect	Description
Level 1: Preplanning	Involvement of stakeholders (decision makers, users, etc.) in the development of telemedicine initiatives	User-centred design processes should involve potential users – in this case patients, citizens, healthcare professionals, etc.
	Holistic objective for telemedicine implementation	Vision for telemedicine in the community should include all stakeholders and areas in the community itself
	Basic technical/ infrastructural requirements are clear to those planning telemedicine initiatives	Instead of needing to know all the technical/ infrastructural requirements from the beginning of the TI onwards, the basic requirements needed to set up telemedicine in the first place (e.g. internet, Bluetooth, and/or telephone line) need to be known
	Risk management	Probability of occurrence and extent of damage for possible risks need to be known and adequately addressed
	Compliance with regulatory requirements	All regulatory requirements need to be clear and followed (e.g. regarding jurisdiction, liability)
	Contractual arrangements are documented	All arrangements are recorded to track agreements (written contracts)
	Ethical guidelines are followed for telemedicine	Guidelines for ethics and governance exist, are known, and followed
	Essential technical infrastructure is provided	Basic hardware and/or software for delivering telemedicine services needs to be provided (e.g. iPad, SIM card)
Level 2: Preparation	Step-by-step implementation plan	Continuous operationalisation of the holistic objective, including the definition of processes, responsibilities and workforce
	Requirements, aims and outcomes for telemedicine initiatives in the community are defined	Expectations for implementing TIs in the community are defined and in line with the community objectives and the implementation plan
	The information management is transparent to all stakeholders	Provision of sufficient information to actively address worries and anxieties
	Awareness campaigns for the existence of TIs to expand knowledge about them	Users need to know about the existence of TIs, otherwise they cannot use them
	Existence of hierarchical structures, which can be adapted for telemedicine responsibilities	This is a prerequisite for the assignment of responsibilities so that things can move forward (the structures should preferably be documented in written form)
	Guidelines for decision making in the community support the community climate and are in line with the beliefs of the community	Hierarchical decision making is possible to avoid missing consensus and conflict potential --> if it violates community values, parallel structures would be created, which hinder efficiency and lead to stubbornness
Level 3: Initiation	Awareness campaigns about the existence of TIs, which also include the users´ peers as target group	Users of TIs can be influenced by their peers
	One or more person(s) are designated as responsible for telemedicine and authority is allocated to them	Based on the existing hierarchical structures, there are clear decision-making competencies (which should be located with existing people to avoid parallel structures and to bundle knowledge/competence)

	Provision of a forum for communication, (inter- and intra-group) cooperation, data management, and the creation of trust among stakeholders who are mutually dependent on each other	Cooperation of the involved actors is directly supported and made transparent for all persons involved (according to community climate and beliefs)
	Provision of adequate financial resources for TIs	The focus here is explicitly on community resources
Level 4: Stabilisation	All technical/ infrastructural requirements are clear to those planning telemedicine initiatives	At this point, all requirements need to be clear to provide the best possible TI
	A sustainable scaling up strategy is defined	Continuous implementation of the vision in the existing system should be combined with a realistic implementation strategy
	Awareness campaigns of benefits of telemedicine are based on effectiveness studies	Information only about the existence of telemedicine is not enough for permanent use; benefits should be shown and communicated as soon as possible
	Provision of adequate human resources for TIs	The focus here is explicitly on community resources
	The communication is culturally appropriate to reach all potential end-users equally	Equal access should be ensured by including all potential users and by a supportive community climate
Level 5: Confirmation/ Expansion	Continuous improvement/ performance management (including supportive policies)	TIs should not be left to itself; once they are up and running, uniform rules for all TIs in the community need to be in place and monitored regularly
	Programs to diminish existing inequalities in healthcare and to increase (health/digital) literacy	As another aspect of inclusion, equal access to using the existing TIs should be ensured for all members of the community (inequalities can result from, e.g., education, socio-economic factors, demographics, or health and employment status)
	Training and qualification for all relevant stakeholders	Increase competence, ability and comfort when using telemedicine and improve (e-) health literacy for all stakeholders (not only frontrunners use and have the chance, but usage hurdles are removed)
Level 6: Professionalisation	Structural programs to ensure that an adequate number of people is available in the workforce and equipped with the necessary skills and knowledge	Proprietary programs for the development of well-trained workforces exist to ensure long-term sustainable use
	Telemedicine initiatives and further existing IT infrastructure are interoperable	The telemedicine initiatives are no isolated solutions that can die off more quickly in favour of an all-encompassing platform that has different compartments

Annex A2 - Adaptions made based on the twelve evaluation interviews

	Category	Adaption	Example quotes/evidence
Assessment	Status of telemedicine initiatives	No adaption necessary	“the first line […] reads very coherently” (SIE7)
	Community involvement	Addition of percentage shares	“where is the border between a small part and the majority? […] there´s no quantification in here” (SIE7); “what is a small part? 10%, 20%, 30%?” (SIE5)
	Evidence for telemedicine in the community	Adaption to current version to increase delimitation between descriptions	
	Level descriptions		
Improvement	Improvement aspects	Assignment to levels (e.g. “risk management”, shifted from level 1 to level 2)	Overall median = 1.5, many experts expressed the view that risks need to be managed from the beginning onwards as, even if small risks occur, they can prevent further success of the telemedicine initiative
		Assignment to levels (e.g. “sustainable scaling up strategy” shifted from level 2 to level 4)	Overall median = 3.5
		Wording (e.g. “all technical/ infrastructural requirements are clear to those planning telemedicine initiatives”, where a target audience for the requirements was added and the aspect was accompanied by “basic technical/ infrastructural requirements are clear to those planning telemedicine initiatives”)	“to whom are they known? […] users […], doctors or developers?” (SIE2)
		Splitting (e.g. “all technical/ infrastructural requirements are clear to those planning telemedicine initiatives” was accompanied by “basic technical/ infrastructural requirements are clear to those planning telemedicine initiatives”)	“’all’ is always such a big word” (SIE1)
		Merging (e.g. of having persons being responsible for telemedicine and allocating authority to them to “one or more person(s) are designated as responsible for telemedicine and authority is allocated to them”	

Annex A3 - Results of assignments from all interviews – the table shows the individual assignment of improvement factors to levels per interview partner

Legend:
"-": The aspect was not rated by the interviewee (e.g. "Basic technical/ infrastructural requirements are clear to those planning telemedicine initiatives" was added over time);
"X.5": The interviewee rated the aspect between two levels

Level	Improvement aspect	Rating per interview partner (Structure interview expert (SIE) no)												Median		
		1	2	3	4	5	6	7	8	9	10	11	12	Total	Australia	Germany
Level 1: Preplanning	Stakeholders (decision makers, users, etc.) are involved in the development of telemedicine initiatives	2	1	2	2	1	1.5	1	1	2	2	2	1	1.75	2	1.5
	Holistic objective for telemedicine implementation	5.5	1	3	2	1	1	3	-	1	1	2	1.5	1.5	1.25	2
	Basic technical/ infrastructural requirements are clear to those planning telemedicine initiatives	-	1	-	-	-	-	-	1	1	2	2	1	1	1	1
	Risk management	1	1	4	3.5	2	4	2	1	1	6	1	1	1.5	1	2
	Compliance with regulatory requirements	1	1	1	3	2	3	4	1	1	6	1	2	1.5	1	2
	Contractual arrangements are documented	1	1	1	3	1	1	4	-	3	6	-	1	1	3	1
	Ethical guidelines are followed for telemedicine	3.5	1	3	3	1	2	-	1	1	1	1	1	1	1	2.5
	Essential technical infrastructure is provided	1	1	1	1	2	3	1	1	2	4	1	2	1	2	1
Level 2: Preparation	The community has a step-by-step implementation plan	2.5	2	4	2	2	3	2	2.5	3	3	4	1.5	2.5	3	2
	Requirements, aims and outcomes for telemedicine initiatives in the community are defined	2.5	2	1	3	2	1.5	2	2	1	1	1	1	1.75	1	2
	The information management is transparent to all stakeholders	2	1	5	2	3	4	2	1	3	2	3	1	2	2	2
	Implementation of awareness campaigns for the existence of telemedicine to expand knowledge about telemedicine initiatives	2	2	4	2	3	1	2	3	1	3	5	1	2	3	2
	Existing hierarchical structures need to be defined clearly to be adapted for telemedicine responsibilities	2	2	1	4	2	1	1	-	1	3	3	1	2	2	2
	The community has guidelines for decision making that support the community climate and are in line with the beliefs of the community	3.5	1	3	3	1	2	2	-	2	2	2	2	2	2	2
Level 3: Initiation	Extension of awareness campaigns by including the users' peers as target group	3	3	5	4.5	3	-	-	3	3	4	4	2	3	3	3
	One or more person(s) are designated as responsible for telemedicine and authority is allocated to them	3.5	2	2	4	2.5	3	2	-	2	1	3	1	2	1.5	2.5

	The community provides a forum for communication, (inter- and intra-group) cooperation, data management, and the creation of trust among stakeholders who are mutually dependent on each other	2	3	6	3	4.5	3	1	-	3	2	3	2	3	2.5	3
	The community provides adequate financial resources for telemedicine initiatives	4	1	6	2	3	3	3	2	3	5.5	1	3	3	3	3
Level 4: Stabilisation	All technical/ infrastructural requirements are clear to those planning telemedicine initiatives	3	-	5	3	2	3	4	5	2	3	6	2	3	3	3
	A sustainable scaling up strategy is defined	3.5	2	3	4.5	4	-	3	4	3	4	3	4	3.5	4	3.25
	Awareness campaigns of benefits of telemedicine exist (based on effectiveness studies)	4	4	6	4.5	2	2	4	5.5	2	2	5	4	4	4	4
	The community provides adequate human resources for telemedicine initiatives	4	1	4	2	3	4	3	3	3	3	2	2	3	3	3
	The communication is culturally appropriate in order to reach all potential end-users	5	3	5	4	2	4	5	5	1	2	2	1.5	3.5	2	4
Level 5: Confirmation/ Expansion	Continuous improvement/ performance management (including supportive policies)	5	2	5	4	6	5	6	3.5	2	6	2	2	4.5	2	5
	The community sets up programs to diminish existing inequalities in healthcare and to increase (health/digital) literacy	5.5	5	5	3	4	6	3	5	3	1	4	2.5	4	3	5
	Training and qualification are available for all relevant stakeholders (to increase competence, ability and comfort when using telemedicine and to improve (e-) health literacy)	5	4	6	5	4	1	4	1	3	3	6	1	4	3	4
Level 6: Profession-alisation	The community set up structural programs to ensure that an adequate number of people is available in the workforce and equipped with the necessary skills and knowledge	6	4	5	6	3	5	6	1.5	3	4	1	3	4	3	5
	Telemedicine initiatives and further existing IT infrastructure are interoperable	2	1.5	5	6	4	2	1.5	5	2	4	2	3	2.5	3	2
